# Effects of intravitreal Bevacizumab (Avastin) monotherapy in central retinal vein occlusion in young subjects

**DOI:** 10.22336/rjo.2024.51

**Published:** 2024

**Authors:** Sarita Lobo, Undrakonda Vivekanand, Geover Joslen Lobo

**Affiliations:** 1 Department of Ophthalmology, Father Muller Medical College, Mangalore, Karnataka, India; 2 Department of Ophthalmology, Alluri Sitaramaraju Academy of Medical Sciences, Maikapuram, Eluri, Andhra Pradesh, India; 3 Department of Neurosurgery, Father Muller Medical College, Mangalore, Karnataka, India

**Keywords:** central retinal venous occlusion, central macular edema, central macular thickness (CMT), best corrected visual acuity (BCVA), BCVA = Best corrected visual acuity, CMT = Central macular thickness, OCT = Optical coherence tomography, IVA = intravitreal Avastin

## Abstract

**Aim:**

To evaluate the efficacy of anti-angiogenic agent Bevacizumab in central retinal venous occlusion treatment.

**Objectives:**

To determine the efficacy of Bevacizumab and ophthalmological parameters such as best corrected visual acuity (BCVA) and central macular thickness (CMT) using optical coherence tomography (OCT) in central retinal venous occlusion in patients aged less than 30.

**Methods:**

This is a prospective, interventional study, done on 25 eyes of 25 patients aged 30 years and below with central retinal venous occlusion, who received intravitreal Bevacizumab injections for three consecutive months. The mean change in best corrected visual acuity (BCVA), and central macular thickness (CMT) measured by optical coherence tomography were compared and correlated at baseline after 3 months and 6 months follow-up.

**Results:**

The mean best corrected visual acuity and the central macular thickness on OCT improved significantly from 1.08±0.29 and 454.80±114.5µm at baseline to 0.77±0.32 logMAR units and 339.7±82.5µm after 6 months follow up.

**Conclusion:**

The current study showed that intravitreal Bevacizumab at a dose of 1.25 mg and with strict control of systemic contributory parameters seemed to improve BCVA and CMT and macular edema measured at baseline to 3 months and 6 months follow-up after three consecutive monthly injections.

## Introduction

One of the causes of blindness globally is venous occlusion-related macular edema.

The prevalence of macular edema (DMO) worldwide is 7.48%. Macular edema is the most common cause of loss of central visual acuity in Central retinal venous occlusion. Macular edema is defined as the edema within 1-disc diameter of the fovea [1]. Although vision loss related to proliferative retinopathy is more common worldwide, CRVO is the second leading cause of macular edema. The major cause of macular edema in CRVO is retinal hypoxia, which, in turn, increases the expression of vascular endothelial growth factor (VEGF). Macular edema (MO) is the thickening of the macula due to fluid accumulation in the extracellular spaces of the retina [2].

Bevacizumab (Avastin) is an inhibitor of the VEGF that is used in diabetic macular edema (DME), age- related macular degeneration (ARMD) and cystoid macular edema (CMO) intravitreally. It is a monoclonal IgG1 antibody produced by recombinant deoxyribonucleic acid (DNA) technology. It binds to VEGF and inhibits the VEGF from binding to its receptors on the surface of endothelial cells, thus neutralizing VEGF and preventing neovascularization [3].

The aim is to determine whether the subsequent response to intravitreal Bevacizumab (IVB) is correlated with pre and post-treatment ocular variables for optical coherence tomography (OCT) and best-corrected visual acuity (BCVA) in eyes with persistent CRVO-related macular edema (DME).

## Methods

The study included patients of age < 30 years with central retinal vein occlusion, macular edema, and OCT evidence of macular edema, who did not undergo laser treatments in the past. The study was conducted from January 2022 to December 2022. Patients of age ≤ 30 years, with other conditions, such as retinal dystrophies and degenerations, glaucoma, optic atrophy due to various causes, and diabetic macular edema, whose fundus could not be assessed due to media opacity, and those who received laser therapy in the past, were excluded from the study.

This was a prospective study, which included a total of 25 eyes of 25 individuals with CRVO and macular edema, attending ophthalmology OPD, and being followed up for 3 and 6 months after the administration of 3 intravitreal Bevacizumab injections for three consecutive months. The institutional research committee and ethics committee approval was obtained. Written informed consent was obtained from each patient after explaining the risk-benefit ratio.

The demographic, medical, and ocular history were obtained. There were no sex-based or racial/ethnic-based differences. Ocular examination including BCVA with Snellen’s chart, slit lamp biomicroscopy, dilated fundus evaluation using a 90D lens, intraocular pressure (IOP) measurement with Goldman applanation, OCT testing using a spectral domain OCT was performed and relevant systemic investigations were done. A treatment plan was composed according to investigations. The procedure of injecting Avastin for all patients was performed in the operating theatre to minimize the risk of endophthalmitis. Pre-operative preparation was done with topical 5% povidone-iodine, 1.25 mg/0.05 ml of Bevacizumab was injected into the vitreous cavity with a 30-gauge needle, 3-4 mm posterior to the inferotemporal limbus. Postoperatively, the patient was put on topical antibiotics for 5 days. The patients then received an injection for three consecutive months from the time of diagnosis. A follow-up was done in the 3rd and 6th months after the final injection. All patients were investigated for the above parameters with a full ocular examination during each visit.

The data were analyzed in Microsoft Excel with Statistical Package for Social Sciences (SPSS) 26 trial version. Baseline characteristics were presented in mean (SD). Comparison of CMT and BCVA between the pre- and post-injection were analyzed with repeated one-way ANOVA. The P < 0.05 was considered significant.

## Results

25 eyes were tested in this study, with a minimum follow-up at the 3rd month, and 6th month after the 3rd injection of Bevacizumab was included for analysis. There were 12 (48%) females and 13 (52%) males with an overall mean age of 25.60±6.67 years. The mean BCVA at baseline was 1.08±0.29 logMar units, at the third-month follow-up it was 0.98±0.34 logMar units, and in the 6th month, it was 0.77±0.32 logMar units. No statistical difference was observed (p = >0.05) between baseline and 3rd month follow up whereas a significant difference (P= <0.05) was observed between 3rd and 6th month (**Table 1**).

**Table 1:** Demographics of all 25 patients at 3rd and 6th month follow-up

	PRE-TREATME NT	AT 3RD MONTH LY FOLLO W UP	AT THE 6TH MONTH FOLLO W UP	SIGNIFICA NCE
VISUAL	1.08±0.29	0.98±0.3	0.77±0.3	< 0.05
ACUITY		4	2	
(LogMAR)				
CMT	454.8±114	401.4±9	320.7±6	< 0.05
(µm)	.5	3.6	2.5	

Laboratory markers were done at each visit.

Optical coherence tomography (OCT) was done for all 25 eyes. At baseline, the mean CMT measurement was 454.8±114.5 µm, improving to 401.48±93.6 µm in the 3rd month and 320.7±72.5 µm in the 6th-month follow-up examination, a significant difference from baseline values (P = <0.05) being shown in **Table 1**.

## Discussion

In this study, 25 eyes, with macular edema secondary to vascular occlusion, were treated with intravitreal Bevacizumab. This resulted in anatomic and functional improvement after 3 consecutive monthly injections, at the 3rd and 6th month follow- ups. BCVA has improved from 1.08±0.2 (6/60 in Snellen’s chart) before the treatment to 0.7±0.3 (6/30 in Snellen chart) and CMT also improved from 454.8±114.5 to 320.7±62.5 at the 6th month follow up. The pathophysiologic mechanism responsible for CRVO is that VEGF causes changes in the tight junctions of retinal endothelium, thus, stimulating the growth of endothelial cells and enhancing vascular permeability, increasing vascular permeability and increasing the macular edema. The introduction of intravitreal anti-VEGF injections has revolutionized the management of macular edema. Increasing the frequency of anti-VEGF injections is thought to benefit even cases with poor vision.

In this study, intravitreal Bevacizumab was given in three consecutive monthly injections, and its efficacy was tested by optical coherence tomography for central macular thickness (CMT) assessment. The best-corrected visual acuity (BCVA) was determined in 25 eyes, inferring a modest change in BCVA between pre-treatment and the 3rd-month follow-up after the last injection, a significant change was observed from the 3rd and 6th-month follow-up. There was a mild improvement in the CMT between pre- treatment and at the 3rd-month follow-up and moderate improvement from the 3rd to 6th-month follow-up.

In a study by Dascalu et al., the medium follow-up period was 9,7 months (6-20 months). There were no significant complications following the procedure. The number of intravitreal Bevacizumab injections varied from 2-5/ patient. All the patients experienced an improvement in VA and a significant regression of macular edema [4]. A prospective study by Kabunga RR et al. [5] assessed the effect of IVA therapy for MO at three months in 32 patients. The study inferred a modest change in CMT and significantly improved BCVA among patients with mainly DMO. The mean CMT presented a significant improvement, and the mean BCVA improved at three months of follow-up after IVA. The improvement in CMT and BCVA was more marked in patients who had diabetic ME compared to other causes.

In the four trials comparing IVB with a comparison group, IVB improved VA and CMT values in the long term (12 weeks) in patients with BRVO- associated macular edema. Furthermore, statistically significant improvements in VA in the short-term (4 weeks) could also be seen [6].

Kartasasmita AS et al. suggested combination therapy of laser photocoagulation and single intravitreal bevacizumab injection resulted in a significantly better visual acuity compared to laser photocoagulation therapy (0.35 versus 0.13 logMAR; *P*=0.041) and reduced macular thickness by 120.33 µm versus 71.50 µm (*P*=0.277), although this difference was not significant [3]. A study by Shalchi Z suggests that treatment of MO secondary to BRVO with anti-VEGF improves visual and anatomical outcomes at six and 12 months [7].

## Conclusion

There are few studies on the short-term effects of Bevacizumab in CRVO unlike in diabetic retinopathy. The limitations of our study are a small sample size and a shorter follow-up period. Also, the macular edema pattern and the integrity of inner and outer retinal layers on OCT were not analyzed. The response to Avastin may differ based on the pattern of macular edema.

The current study showed that the advent of anti- VEGF therapy and strict control of systemic contributory factors that interfere with the therapeutic response would present visual (BCVA) and anatomical (CMT) parameters improvement.
